# Antiplatelet therapy for *Staphylococcus aureus* bacteremia: Will it stick?

**DOI:** 10.1371/journal.ppat.1010240

**Published:** 2022-02-10

**Authors:** Alexander M. Tatara, Ronak G. Gandhi, David J. Mooney, Sandra B. Nelson

**Affiliations:** 1 Division of Infectious Diseases, Massachusetts General Hospital, Boston, Massachusetts, United States of America; 2 Wyss Institute for Biologically Inspired Engineering, Harvard University, Boston, Massachusetts, United States of America; 3 Harvard John A. Paulson School of Engineering and Applied Sciences, Harvard University, Cambridge, Massachusetts, United States of America; 4 Department of Pharmacy, Massachusetts General Hospital, Boston, Massachusetts, United States of America; Nanyang Technological University, SINGAPORE

## Abstract

*Staphylococcus aureus* bacteremia (SAB) remains a clinically challenging infection despite extensive investigation. Repurposing medications approved for other indications is appealing as clinical safety profiles have already been established. Ticagrelor, a reversible adenosine diphosphate receptor antagonist that prevents platelet aggregation, is indicated for patients suffering from acute coronary syndrome (ACS). However, some clinical data suggest that patients treated with ticagrelor are less likely to have poor outcomes due to *S*. *aureus* infection. There are several potential mechanisms by which ticagrelor may affect *S*. *aureus* virulence. These include direct antibacterial activity, up-regulation of the innate immune system through boosting platelet-mediated *S*. *aureus* killing, and prevention of *S*. *aureus* adhesion to host tissues. In this Pearl, we review the clinical data surrounding ticagrelor and infection as well as explore the evidence surrounding these proposed mechanisms of action. While more evidence is needed before antiplatelet medications formally become part of the arsenal against *S*. *aureus* infection, these potential mechanisms represent exciting pathways to target in the host/pathogen interface.

## Author summary

*Staphylococcus aureus* remains a challenge to treat given its virulence and its ability to invade the bloodstream and spread to multiple sites in the body. Recently, it has been observed that patients taking the antiplatelet medication ticagrelor may have better infection outcomes. From this clinical observation, investigators have launched in vitro and animal studies to better understand by which mechanisms ticagrelor may affect *S*. *aureus* infection and clearance. In this Pearl, we review clinical data surrounding ticagrelor and infection as well as explore 3 different potential mechanisms of action that have been suggested by current studies. These mechanisms may involve boosting the host’s platelet-mediated innate immunity, representing an exciting direction for the treatment of *S*. *aureus* bacteremia.

## Introduction

*Staphylococcus aureus* bacteremia (SAB) remains a major clinical challenge with significant patient morbidity and mortality. To better address SAB, investigators seek antibacterial strategies that act in nontraditional ways, including those that augment the host immune response [1]. While platelets are well known for their role in thrombosis, they also participate in innate immunity. In vitro, platelets successfully kill *S*. *aureus* [2]. Platelets can phagocytose *S*. *aureus* as well as secrete antibacterial peptides from alpha granules that kill *S*. *aureus* independent of antibodies [3,4]. In addition to direct activity against *S*. *aureus*, platelets can also be activated by intravascular pathogens due to pattern recognition receptors, causing secretion of chemokines to recruit and enhance lymphocytes as well as communicate with endothelial cells [2,5], thereby augmenting the immune response. Clinically, thrombocytopenia in the setting of SAB has been associated with both a greater magnitude of bacteremia and patient mortality [6], although it is not clear if this relationship is correlative or causative.

With advances in vascular medicine, platelet-modifying drugs such as ticagrelor, clopidogrel, and prasugrel are often prescribed for up to 1 year to patients suffering from acute coronary syndrome (ACS) [7]. In the Study of Platelet Inhibition and Patient Outcomes (PLATO) randomized controlled trial [7], ticagrelor was found to be superior to clopidogrel in preventing death from myocardial infarct, stroke, and vascular causes in patients with ACS. Ticagrelor is a reversible inhibitor of the platelet adenosine diphosphate P2Y_12_ receptor, whereas clopidogrel and prasugrel are irreversible inhibitors of the same receptor. It remains unclear whether platelet-modifying therapeutics influence the role of platelets in innate immunity, although there is preliminary in vitro, in vivo, and clinical evidence that ticagrelor may mitigate SAB.

Here, we review clinical evidence surrounding ticagrelor and infection as well as explore 3 potential pathways in which ticagrelor may inhibit *S*. *aureus*.

### Clinical data suggest that ticagrelor alters infection outcomes compared to patients taking other antiplatelet medications

In the PLATO trial, over 18,000 patients with ACS were treated with 1 year of either ticagrelor or clopidogrel [7]. In a post hoc analysis of patients, adverse events were studied including rates of bacteremia/sepsis [8]. Although the rates of these infections were similar in both groups, there were fewer deaths due to sepsis/bacteremia in the ticagrelor group (7 versus 23; *p* = 0.003).

The PLATO study renewed interest regarding infectious outcomes in patients following ACS. A total of 3 retrospective studies were published comparing patients on clopidogrel and ticagrelor. Among 9,518 patients treated with ticagrelor or clopidogrel (matched using propensity scoring), there were significantly fewer hospital readmissions due to infection with ticagrelor (6.11%) than with clopidogrel (10.53%) (HR 0.736, 95% CI 0.64 to 0.85; *p* < 0.001) [9]. In another propensity-matched retrospective study, 1.4% of 1,356 patients treated with ticagrelor compared to 3.6% of 1,356 patients treated with clopidogrel had gram-positive infections (HR 0.37; 95% CI 0.22 to 0.63; *p* < 0.001) [10]. Last, a third retrospective study including over 26,000 patients measured the occurrence of SAB during the first year after initiation of either ticagrelor or clopidogrel [11]. Patients treated with ticagrelor had significantly fewer episodes of SAB with absolute risk reduction of −0.19% (95% CI −0.32% to −0.05%; *p* = 0.006).

Notably, the findings from PLATO were a post hoc analysis, and these retrospective studies were correlative and not designed to determine cause and effect. However, these data in sum suggest that there may be mechanisms by which ticagrelor mitigates infection risk and, potentially, SAB.

### Ticagrelor has direct activity against *S*. *aureus*, albeit at supraphysiologic concentration

When evaluated with in vitro time–kill assays, ticagrelor was effective against methicillin-resistant *S*. *aureus* (MRSA), methicillin-susceptible *S*. *aureus* (MSSA), *Staphylococcus epidermidis*, *Streptococcus agalactiae*, and *Enterococcus faecalis* [12]. It was not effective against 2 gram-negative pathogens, *Escherichia coli* and *Pseudomonas aeruginosa*. However, its antibacterial activity occurred at supraphysiologic concentrations. The minimum inhibitory concentration (MIC) of ticagrelor against MRSA was 20 μg/mL, whereas the physiologic concentration of ticagrelor at dosing for ACS in humans is between 0.8 and 1.2 μg/mL [12]. Another study also found that ticagrelor inhibited a clinical isolate of MSSA but only at supraphysiologic concentrations (MIC 64 μg/mL) [13]. Further, the combination of ticagrelor with the antimicrobials cefazolin or ertapenem was only additive rather than synergistic.

If concentrations of ticagrelor required for direct antistaphylococcal activity are not achievable clinically, how does ticagrelor exert an antibacterial effect at physiologic doses? In a murine model in which MRSA-inoculated polyurethane disks were implanted in the flanks of immunocompetent mice, those treated with ticagrelor at physiologic dosing had significantly decreased bacterial burden of their infected implant, suggesting that another mechanism in vivo may be driving the antistaphylococcal activity of ticagrelor [12]. In sum, these results indicate that direct antibacterial activity of ticagrelor is unlikely to account for its apparent activity in vivo.

### Ticagrelor improves host platelet–mediated killing of *S*. *aureus* and decreases host thrombocytopenia

Platelets engage in the clearance of *S*. *aureus* by secretion of antimicrobial peptides and phagocytosis of bacteria as well as recruitment of other lymphocytes [2–5]. In vitro, ticagrelor at physiologic concentration significantly enhanced the ability of human platelets to kill MRSA, whereas aspirin (another antiplatelet drug) did not [14]. The same effect was reproducible against MSSA [13]. Under microscopy, platelets incubated with *S*. *aureus* developed significant structural damage, although platelets treated with ticagrelor were relatively preserved, suggesting that ticagrelor may have a protective/stabilizing effect on platelets in the setting of *S*. *aureus* exposure [14].

In an observational prospective study of 49 consecutive patients with SAB, thrombocytopenia correlated with increased mortality [14]. Notably, isolates from SAB patients with more severe thrombocytopenia produced more α-toxin [14], an exotoxin that increases hepatic clearance of platelets through platelet desialylation. Mice infected with α-toxin–deficient *S*. *aureus* mutants had decreased thrombocytopenia and bacterial burden compared with mice infected with wild-type *S*. *aureus*. However, mice pretreated with physiologic concentrations of ticagrelor had decreased thrombocytopenia and improved survival during wild-type SAB [14].

In a clinical case report, a 60-year-old man with refractory SAB and thrombocytopenia despite 5 days of antibiotic therapy was started on ticagrelor [13]. Within 24 hours, his bacteremia resolved and platelet count improved. Discontinuation of ticagrelor led to recurrent thrombocytopenia, which then reversed with the resumption of ticagrelor. The patient was treated with 3 months of ticagrelor in addition to standard antibiotic therapy without further infection recurrence.

In sum, these in vitro and in vivo studies suggest that ticagrelor can enhance platelet-mediated killing of *S*. *aureus* as well as mitigate *S*. *aureus*–induced thrombocytopenia likely by preventing α-toxin–related desialylation. Given the role of platelets in innate immunity, maintaining platelet counts may contribute to improved outcomes in SAB as an additional benefit to ticagrelor therapy.

### Antiplatelet therapy inhibits *S*. *aureus* binding to host endothelial tissues

Among other adherence mechanisms, *S*. *aureus* binds platelets via interactions between its clumping factor A (*clfA*) and host platelet von Willebrand factor and fibrinogen [15]. Activated platelets bind to the exposed extracellular matrix of damaged host endovascular tissue (such as heart valves). Therefore, preventing platelet aggregation on host endothelium by inhibiting platelet activation may mitigate SAB and its infectious complications. For example, *S*. *aureus* mutants lacking *clfA* were 50% less likely to cause endocarditis than wild-type strains in a SAB rat model [16].

In ex vivo perfusion reactors, precoating bovine jugular veins with fibrinogen stimulated both human platelet and *S*. *aureus* surface binding [17]. However, the platelet α_IIb_β3 antagonist eptifibatide decreased *S*. *aureus* adhesion, likely due to inhibition of platelets. Likewise, the effect of antiplatelet therapy with aspirin and ticagrelor on *S*. *aureus* adhesion in the presence of human blood (including platelets) was tested under shear conditions [17]. Treatment with both aspirin and ticagrelor independently decreased *S*. *aureus* attachment to the lumen of bovine jugular veins; the combination of the 2 resulted in significantly less adhesion than aspirin alone. Dual antiplatelet therapy was also found in vivo to decrease endocarditis in a rat model of SAB due to inhibition of platelet binding [18]. Preventing *S*. *aureus* binding to platelets and therefore minimizing contact with host tissues may be another mechanism by which ticagrelor and other antiplatelet drugs mitigate infection.

## Future directions

**Fig 1:**
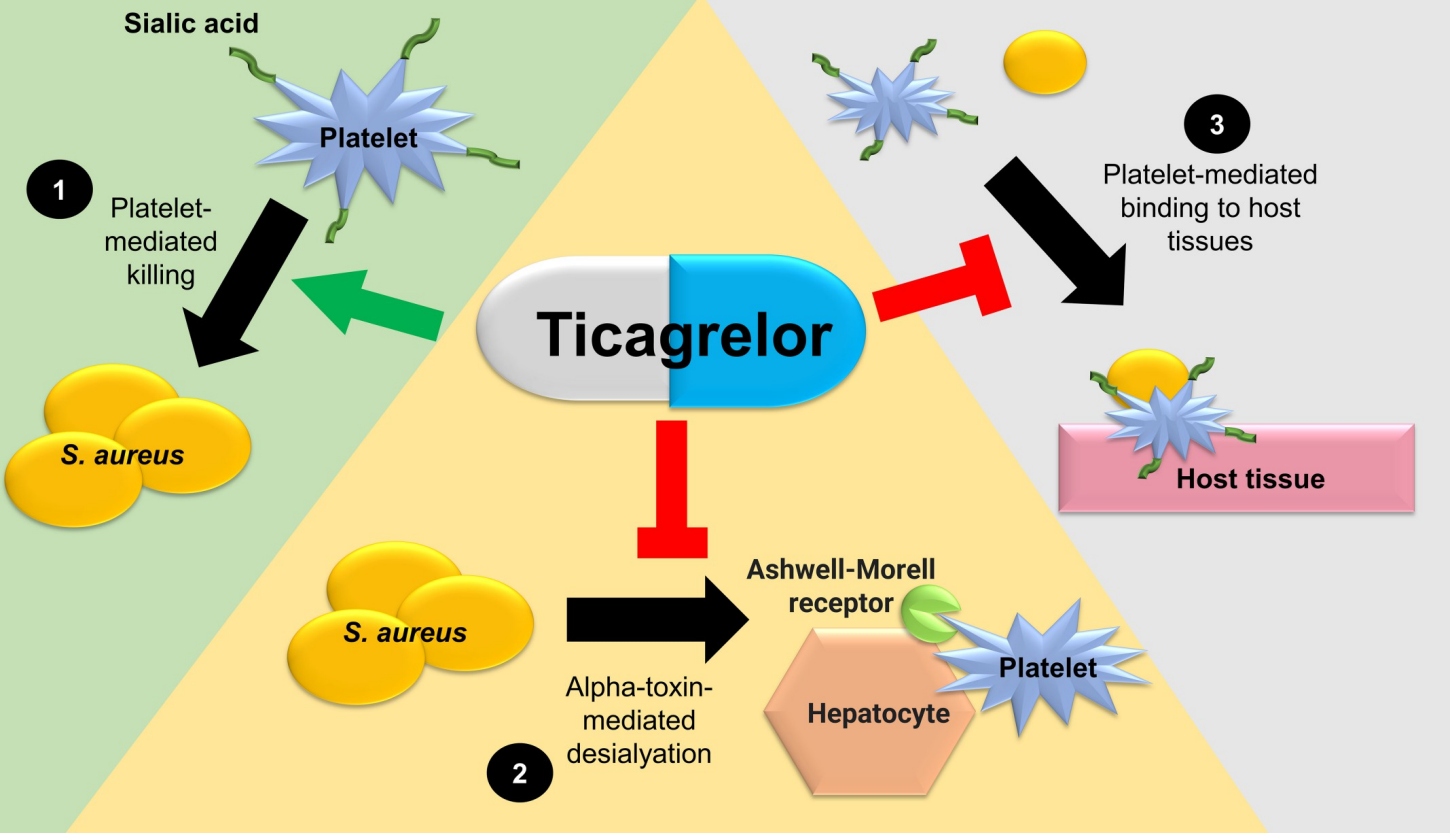
Schematic of proposed pathways in which ticagrelor may affect *S*. *aureus* infection (not to scale): (1) enhancement of platelet-mediated *S*. *aureus* killing; (2) prevention of α-toxin–mediated platelet desialyation and subsequent removal from circulation via the hepatic Ashwell–Morell receptor; and (3) inhibition of *S*. *aureus* binding to host tissues via adhered platelets.

Clinical data from large patient cohorts suggest a protective effect of ticagrelor against infection. In vitro and rodent models have demonstrated that ticagrelor has direct antistaphylococcal activity at high concentrations and facilitates platelet-mediated killing of *S*. *aureus*, decreases SAB-induced thrombocytopenia, and mitigates binding of *S*. *aureus* to platelets and host tissue at physiologic concentrations (Fig 1). Therapeutic strategies that improve host immune function are appealing, as these are not prone to traditional bacterial resistance mechanisms [1]. In addition, repurposing existing licensed drugs is attractive, as the safety and adverse event profiles are well documented [19].

Stronger evidence is needed to conclusively evaluate the clinical efficacy of ticagrelor in SAB. A prospective randomized controlled trial of patients receiving standard of care versus standard of care plus ticagrelor may bring further clarity. Anticipated risks and unanticipated consequences, including increased bleeding, would need to be carefully considered. In a prospective trial of over 20,000 patients, those randomized to take low- or high-dose ticagrelor did have significantly greater bleeding compared to placebo (6.2, 7.8, and 1.5%, respectively; *p* < 0.01), although 86% of bleeding events were nonmajor [19]. Furthermore, there are conflicting reports that patients with endocarditis on anticoagulation may be more prone to cerebral hemorrhage due to emboli [20], and this will further need to be weighed as a potential risk with use of ticagrelor in SAB. While promising, the potential of antiplatelet medication to treat staphylococcal infection remains uncertain.
